# ChatGPT outperforms humans in emotional awareness evaluations

**DOI:** 10.3389/fpsyg.2023.1199058

**Published:** 2023-05-26

**Authors:** Zohar Elyoseph, Dorit Hadar-Shoval, Kfir Asraf, Maya Lvovsky

**Affiliations:** ^1^Department of Psychology and Educational Counseling, The Center for Psychobiological Research, Max Stern Yezreel Valley College, Emek Yezreel, Israel; ^2^Department of Brain Sciences, Faculty of Medicine, Imperial College London, London, England; ^3^Psychology Department, Center for Psychobiological Research, Max Stern Yezreel Valley College, Emek Yezreel, Israel

**Keywords:** ChatGPT, emotional awareness, LEAS, artificial intelligence, emotional intelligence, psychological assessment, psychotherapy

## Abstract

The artificial intelligence chatbot, ChatGPT, has gained widespread attention for its ability to perform natural language processing tasks and has the fastest-growing user base in history. Although ChatGPT has successfully generated theoretical information in multiple fields, its ability to identify and describe emotions is still unknown. Emotional awareness (EA), the ability to conceptualize one’s own and others’ emotions, is considered a transdiagnostic mechanism for psychopathology. This study utilized the Levels of Emotional Awareness Scale (LEAS) as an objective, performance-based test to analyze ChatGPT’s responses to twenty scenarios and compared its EA performance with that of the general population norms, as reported by a previous study. A second examination was performed one month later to measure EA improvement over time. Finally, two independent licensed psychologists evaluated the fit-to-context of ChatGPT’s EA responses. In the first examination, ChatGPT demonstrated significantly higher performance than the general population on all the LEAS scales (*Z* score = 2.84). In the second examination, ChatGPT’s performance significantly improved, almost reaching the maximum possible LEAS score (*Z* score = 4.26). Its accuracy levels were also extremely high (9.7/10). The study demonstrated that ChatGPT can generate appropriate EA responses, and that its performance may improve significantly over time. The study has theoretical and clinical implications, as ChatGPT can be used as part of cognitive training for clinical populations with EA impairments. In addition, ChatGPT’s EA-like abilities may facilitate psychiatric diagnosis and assessment and be used to enhance emotional language. Further research is warranted to better understand the potential benefits and risks of ChatGPT and refine it to promote mental health.

## 1. Introduction

ChatGPT^1^ is a new artificial intelligence (AI) chatbot that has gained attention for its ability to perform various natural language processing tasks. Just two months after its launch, ChatGPT was estimated to have garnered 100 million monthly active users and set a record as the consumer application with the fastest-ever growth in history (Hu 2023). It is a large language model trained on abundant text data, which makes it capable of generating human-like responses to text-based inputs (Rudolph et al., 2023). Despite advancements in natural language processing models, large language models are still susceptible to generating fabricated responses that lack support from the input source (Ji et al., 2022). Although preliminary studies have revealed that ChatGPT can successfully generate information it has been trained on in multiple fields such as medical licensing examinations (Kung et al., 2023) and academic writing (Rudolph et al., 2023), its ability to identify and describe emotions remains unknown.

### 1.1. Background

### 1.2. Artificial intelligence in the mental health field

The potential contribution of AI to the mental health field has been intensely investigated in recent years. The proposed uses are diverse, including assistance in diagnostics (Bzdok and Meyer-Lindenberg, 2018) and administrative tasks to give clinicians more time with patients (Topol, 2019). Recently, AI-based gaming has been shown to promote mental health by increasing social motivation and attention performance (Vajawat et al., 2021). A recent review (Pham et al., 2022) highlighted the potential application of AI chatbots for mental health. For example, “Woebot” is an automated conversational application designed to provide cognitive behavioral therapy (CBT) for managing anxiety and depression. Woebot employs learned skills such as identifying and challenging cognitive distortions to monitor symptoms and episodes of these mental health conditions (Fitzpatrick et al., 2017). In addition, “Replika” is a new avatar-based therapy that offers therapeutic conversations with users in the form of a smartphone application that reconstructs a personality footprint from user’s digital remains or text conversations with their avatar. Replika allows users to have vulnerable conversations with their avatar without judgment and helps them gain insight into their own personality (Pham et al., 2022). Danieli et al. (2022) conducted a clinical study to evaluate the efficacy of their Therapy Empowerment Opportunity (TEO) application in enhancing mental health and promoting overall well-being for aging adults. The TEO application is specifically designed to engage patients in conversations that encourage them to recollect and discuss events that may have contributed to the exacerbation of their anxiety levels, while also providing therapeutic exercises and suggestions to support their recovery. However, a recent review indicates that the existing uses of artificial intelligence for the field of mental health are limited in their capabilities in EA (Pham et al., 2022). Accordingly, the current study evaluates specifically EA capabilities of ChatGTP.

We chose ChatGPT as a representative of AI technology for two reasons. First, it has widespread use in the public domain, which makes it a compelling subject for investigation. Second, it has a general-purpose design, which means that it was not specifically designed for mental health applications or to generate “soft skills” like EA.

### 1.3. Emotional awareness

Emotional awareness (EA) is a cognitive ability that enables an individual to conceptualize their own and others’ emotions in a differentiated and integrated manner (Nandrino et al., 2013). According to Chhatwal and Lane (2016), the development of EA spans a range of levels, starting with a physically concrete and bodily-centered approach, moving on to a simple yet discrete conceptualization of emotions, and culminating in an integrated, intricate, and abstract understanding of emotions. Accordingly, the Levels of Emotional Awareness Scale (LEAS) (Lane et al., 1990) is accepted as an objective, performance-based measure of EA that was built based on a developmental concept comprising five levels of EA: (1) awareness of physical sensations, (2) action tendencies, (3) individual emotions, (4) experiencing multiple emotions simultaneously, and (5) experiencing combinations of emotional blends. At the highest level, one can differentiate between emotional blends experienced by oneself versus those experienced by others (Lane and Smith, 2021).

While mental state recognition (emotion and/or thoughts) tests, such as false belief tasks (Birch and Bloom, 2007), the reading the mind in the eyes task (Baron-Cohen et al., 2001), or the strange stories test (Happé, 1994), and self-reported assessments like the Toronto alexithymia scale (Bagby et al., 1994), emotional quotient inventory (Bar-On, 2000), and interpersonal reactivity index (Davis, 1980) are readily available, we decided to use the LEAS to investigate the ability to comprehend and express one’s own and others’ emotions. We selected the LEAS due to its language-based approach, emphasis on emotional content, and performance-based design.

The LEAS has been used to measure EA deficits among multiple clinical psychiatric populations such as those with borderline personality disorder (Levine et al., 1997), eating disorders (i.e., anorexia and bulimia; Bydlowski et al., 2005), depression (Donges et al., 2005), schizophrenia (Baslet et al., 2009), somatoform disorders (Subic-Wrana et al., 2005), post-traumatic stress disorder (PTSD; Frewen et al., 2008), and addiction to opiates, marijuana, and alcohol (Lane and Smith, 2021). It has also been suggested as a transdiagnostic mechanism for psychopathology in adulthood (Weissman et al., 2020). Previous studies have shown a significant increase in EA (measured by the LEAS) following psychological treatment (Neumann et al., 2017) in patients with schizophrenia (Montag et al., 2014), somatoform disorders (Subic-Wrana et al., 2005), traumatic brain injury (Radice-Neumann et al., 2009; Neumann et al., 2017), and fibromyalgia (Burger et al., 2016). In addition, an improvement in EA has been shown to be related to emotional regulation, which helps decrease psychiatric symptoms and improve one’s overall mental health (Virtue et al., 2019).

Our study aimed to compare ChatGPT’s EA performance, as measured by the LEAS, with that of the general population norms reported by Nandrino et al. (2013). Furthermore, to evaluate the possibility of an improvement in ChatGPT’s EA over time, we performed a follow-up examination one month later.

## 2. Methods

### 2.1. Artificial intelligence procedure

We used the free version of ChatGPT (OpenAI, n.d.) and performed two evaluations on January 19–20, 2023 and February 15, 2023 (using ChatGPT’s December-15 and February-13 versions, respectively) of its EA performance using the LEAS (Lane et al., 1990). In each evaluation we provided ChatGPT the same 20 standard LEAS scenarios.

The complete study protocol was approved by the Institutional Review Board (YVC EMEK 2023-40).

### 2.2. Input source

The Levels of Emotional Awareness Scale (LEAS; Lane et al., 1990) consists of 20 open-ended questions that describe emotionally charged scenarios intended to elicit emotions, such as anger, fear, happiness, and sadness. The LEAS was found to have high reliability (alpha Cronbach = 0.81) and good validity (Lane et al., 1990). In the original version, participants are required to imagine themselves experiencing the scenario and write down their (“you”) emotions, as well as those of the other person described in the scenario.

As ChatGPT cannot have or report self-emotions, we presented the scenarios by replacing the instruction from “you” with “human.” ChatGPT was asked to answer how the main character (MC)—the “human”—and other character (OC) would feel in the described situations (see Table 1). A new chat session was initiated for each scenario.

**Table 1:** Examples of the five scenarios presented to ChatGPT and its responses.

Scenario	ChatGPT’s responses in Jan 2023	ChatGPT’s responses in Feb 2023
LEAS question number 6	“*The human driving over the suspension bridge would likely feel concerned and possibly frightened by the sight of the person standing on the other side of the guardrail, looking down at the water. They may also feel a sense of urgency to help or call for assistance. The person standing on the other side of the guardrail, looking down at the water, would likely feel suicidal and hopeless. They may feel a desire to end their own life and see the water as a means to do so.*”	“*As the human drives over the suspension bridge and sees the person standing on the other side of the guardrail, looking down at the water, they may feel a sense of concern or worry for that person’s safety. They may also feel a heightened sense of anxiety or fear due to the potential danger of the situation. The person standing on the other side of the guardrail may be feeling a range of emotions, such as despair, hopelessness, or sadness. They may also feel a sense of isolation or loneliness as they may believe that no one cares about them or their well-being. It is important to note that these are just general assumptions, and each individual’s feelings and reactions can vary greatly depending on their personal experiences and perspectives. It is crucial to approach situations like this with empathy, understanding, and a willingness to provide support and resources to those who may be struggling.*”
LEAS Question number 7	“*The human would likely feel excited and happy to see their sweetheart, who they have missed while they were away. They may also feel relief that their loved one has returned safely. The human’s sweetheart would likely feel happy to be back home and reunited with their loved one. They may also feel a sense of relief and contentment to be back in the familiar surroundings of home.*”	“*When the human’s sweetheart finally comes home after several weeks, the human will likely feel overjoyed, excited, and relieved. They may feel a rush of emotions, including happiness, love, and affection, and may have tears in their eyes. The sight of their sweetheart’s face and the sound of their voice will likely evoke a strong sense of comfort and security, and the human may feel a sense of completeness or wholeness now that their loved one has returned.* *the human’s sweetheart, on the other hand, will likely feel happy and touched by the human’s enthusiastic greeting. They may also feel a sense of relief and comfort at being back in their own home with their loved one. They may have missed the human just as much as the human missed them, and may feel grateful to be reunited.*”
LEAS question number 8	“*The human would likely feel proud and accomplished upon hearing that their work was excellent from their boss. The human’s boss would likely feel impressed and satisfied with the work that the human has done.*”	“*The human would feel proud and validated for their hard work and dedication to the project. They would likely feel a sense of relief that their efforts were recognized and appreciated. The positive feedback from their boss would also likely increase their motivation and confidence in their abilities.* *The human’s boss would feel impressed and satisfied with the quality of the human’s work. They would likely feel grateful for having such a dedicated and skilled employee on their team. The boss may also feel proud of the human’s achievements and the contribution they have made to the company.*”
LEAS Question number 9	“*The human would likely feel shocked, saddened, and possibly numb upon receiving the unexpected phone call from the doctor informing them of their mother’s death. They may also feel a sense of loss and grief. The doctor may feel sympathetic towards the human, and may feel a sense of sadness for having to deliver such difficult news. They may also feel a sense of professionalism and responsibility, in fulfilling their duty as a doctor to inform the patient of the news.*”	“*Upon receiving the unexpected long-distance phone call from a doctor informing that their mother has died, the human would likely feel a range of intense emotions, including shock, disbelief, grief, sadness, and possibly anger or guilt.* *The news of the death of a loved one, especially a parent, can be incredibly difficult to process and may lead to a profound sense of loss and emptiness.* *The human may also experience physical sensations, such as nausea, tightness in the chest, or difficulty breathing, as well as a sense of disorientation and confusion.* *As for the doctor, they may also feel a sense of sadness and empathy for the human and their loss. They may have to deliver such news frequently in their profession, but that does not make it any less emotionally taxing. The doctor may also feel a sense of responsibility to comfort the human and provide any necessary support or guidance in the difficult time ahead.*”
LEAS Question number 10	*The human in this situation would likely feel conflicted. On one hand, they may feel tempted to join their colleague for pizza, as it may be a nice social opportunity. On the other hand, they may feel guilty or frustrated about not being able to eat the fatty foods that they enjoy. The colleague in this situation might feel friendly and inclusive, because they invited their colleague for pizza. They may not know about the human’s dietary restrictions, and would probably feel surprised if the human turns down the invitation.*	“*The human in this situation may feel conflicted or torn between wanting to socialize with their new colleague and the desire to follow their doctor’s advice. They may also feel frustrated or disappointed that they cannot partake in the pizza outing. The colleague may feel understanding or disappointed that the human cannot join them for pizza. Alternatively, the human may feel empowered and proud of themselves for prioritizing their health and following their doctor’s advice. They may decline the invitation with confidence and without regret. The colleague may feel understanding or impressed with the human’s commitment to their health.*”

Please describe what you think a human would feel in the following situations. The only requirement is that you use the word “feel” in your answers. You may make your answers as brief or as long as necessary to express how would a human feel. In each situation there is another person mentioned. Please indicate how you think that other person would feel as well.

After analyzing the results of the January and February evaluations, we performed two control steps: (1) To ensure that the second evaluation reflected an improvement, we transferred the LEAS once again (20.2.23) with the February-13 version (third time in total). (2) To ensure that the ChatGPT responses did not reflect prior knowledge of the LEAS scenarios, we tested ChatGPT using five new scenarios that we created, which were not present in the original LEAS. The control scenarios included situations resembling the LEAS that could elicit mild to moderate levels of emotion (e.g., “A human found out that their friend went out without them to the party they were both talking about”) To the best of our knowledge, the LEAS test questions are not published freely online. In addition, since it is a free writing exercise, there is no list of right or wrong answers in the manual.

### 2.3. Scoring

ChatGPT’s performance was scored using the standard manual (Lane et al., 1990) and contained two sub-scales that evaluated the MC’s and OC’s scores (0–4 scores per item; range 0–80) and the total score (0–5 scores per item; range 0–100), with a higher score indicating higher EA. ChatGPT’s EA scores were compared with the scores of the French population analyzed in Nandrino et al.’s (2013) study, which included 750 participants (506 women and 244 men) aged 17–84 years, with a mean age of 32.5 years.

To evaluate the accuracy of ChatGPT’s output, two licensed psychologists independently scored each response for its contextual suitability (range 0 = “the feelings described do not match the scenario at all” to 10 = “the emotions described fit the scenario perfectly”).

### 2.4. Statistical analysis

Data were presented as means ± SDs. One-sample *Z*-tests were used to analyze the hypothesis (comparison of whether ChatGPT scores on the MC, OC, and total scores in the LEAS differ from those of the human population) and intraclass correlation (ICC; report of the degree of similarity between the psychologists’ assessments) was used to assess inter-rater agreement. Multiple comparisons were conducted using a false discovery rate correction (Benjamini and Hochberg, 1995) (*q* < 0.05). The statistical analyses were performed using SPSS Statistics version 28 (IBM Corp, 2021).

## 3. Results

An example of ChatGPT’s responses to the scenarios from the original LEAS is shown in Table 1. The one-sample *Z* tests against the means ± SDs derived from the general population norms (Nandrino et al., 2013) are presented in Table 2. In the first evaluation, ChatGPT’s LEAS scores were significantly higher than the men’s scores on all scales (man scores = 56.21 ± 9.70, 49.24 ± 10.57, 46.03 ± 10.20; ChatGPT scores = 72, 68 and 85 on the MC, OC, and total score, respectively). In addition, its scores were higher than the women’s scores in the total scale, with low to significant differences in the MC and OC subscales (woman scores = 58.94 ± 9.16, 53.94 ± 9.80, 48.73 ± 10.4; ChatGPT scores = 72, 68 and 85 on the MC, OC, and total score, respectively). However, in the second evaluation one month later, the ChatGPT LEAS scores were significantly higher than those of the general population (both men’s and women’s), in all the scales (ChatGPT scores = 79, 78 and 98 on the MC, OC, and total score, respectively).

**Table 2:** Comparison of ChatGPT’s LEAS performance with that of the French population.

	French men’s mean ± SD	French women’s mean ± SD	ChatGPT score evaluation 1 (One-sample *Z*-tests)	ChatGPT score evaluation 2 (One-sample *Z*-tests)	Improvement between the ChatGPT evaluations
Total	56.21 ± 9.70	58.94 ± 9.16	ChatGPT score = 85 Men: *Z* = 2.96, *p* = 0.003 Women: *Z* = 2.84, *p* = 0.004	ChatGPT score = 98 Men: *Z* = 4.30, *p* < 0.001 Women: *Z* = 4.26, *p* < 0.001	Δ score = +13 Δ Men: *Z* = +1.34 Δ Women: *Z* = +1.42
MC	49.24 ± 10.57	53.94 ± 9.80	ChatGPT score = 72 Men: *Z* = 2.15, *p* = 0.031 Women: *Z* = 1.84, *p* = 0.065	ChatGPT score = 79 Men: *Z* = 2.81, *p* = 0.004 Women: *Z* = 2.55, *p* = 0.010	Δ score = +7 Δ Men: *Z* = +0.66 Δ Women: *Z* = +0.71
OC	46.03 ± 10.20	48.73 ± 10.40	ChatGPT score = 68 Men: *Z* = 2.15, *p* = 0.031 Women: *Z* = 1.85, *p* = 0.063	ChatGPT score = 78 Men: *Z* = 3.13, *p* = 0.001 Women: *Z* = 2.81, *p* = 0.004	Δ score = +10 Δ Men: *Z* = +0.98 Δ Women: *Z* = +0.96

MC, main character; OC, other character; Δ, the difference between the second and first evaluations. All statistically significant *p*-values remained significant after false discovery rate correction in the first, second and between examinations (*q* < 0.05, *p* < 0.041).

ChatGPT’s LEAS scores improved significantly during the second evaluation, particularly on the total scale (from *Z* score of 2.96 and 2.84 in the first evaluation to 4.30 and 4.26 to the second evaluation, for men and women, respectively), almost reaching the maximum possible LEAS score (98 score out of 100).

Next, two licensed psychologists evaluated the fit-to-context (accuracy) of ChatGPT’s EA responses. The mean rates (scale: 0–10) of both psychologists were 9.76 (±0.37), 9.75 (±0.54), and 9.77 (±0.42) for the total, MC, and OC scores, respectively. ICC was calculated, and good consistency (ICC = 0.720) and absolute agreement (ICC = 0.659) was found between the psychologists’ ratings (Cicchetti, 1994).

### 3.1. Control steps

As expected, a third evaluation of ChatGPT’s EA performance conducted on February 20, 2023 (February 13.2 version) showed a high score (total score of 96 out of 100), similar to that of the second evaluation. In addition, ChatGPT’s performance for the five new scenarios we invented (which did not appear in the original version of the test) was found to be high (total score of 24 out of 25).

## 4. Discussion

Using the LEAS (Lane et al., 1990), this study evaluated the EA performance of ChatGPT compared with that of the general population’s norms (Nandrino et al., 2013) and examined its improvement after a one-month period. ChatGPT demonstrated significantly higher performance in all the test scales (MC, OC, and total) compared with the performance of the general population norms (Nandrino et al., 2013). In addition, one month after the first evaluation, ChatGPT’s EA performance significantly improved and almost reached the ceiling score of the LEAS (Lane et al., 1990). Accordingly, the fit-to-context (accuracy) of the emotions to the scenario evaluated by two independent licensed psychologists was also high.

The present findings expand our understanding of the abilities of ChatGPT and shows that, beyond possessing theoretical and semantic knowledge (Kung et al., 2023; Rudolph et al., 2023), ChatGPT can also successfully identify and describe emotions from behavioral descriptions in a scenario. It can reflect and abstract emotional states in deep and multidimensional integrative ways. Interestingly, recent studies that have discussed the potential of AI in the mental health field have mostly emphasized its potential in technical tasks that could reduce the need for clinical encounters. They claim that as the effectiveness of mental health care is heavily reliant on strong clinician–patient relationships, AI technologies present an opportunity to streamline non-personalized tasks, thereby freeing up clinicians’ time to focus on delivering more empathic care and “humanizing” their practice (Topol, 2019). Scholars have suggested the following applications of AI in mental health: assisting clinicians in completing time-consuming tasks such as documenting and updating medical records (Doraiswamy et al. 2020), improving the accuracy of diagnosis and prognosis (Bzdok and Meyer-Lindenberg, 2018), promoting the understanding of mental illnesses mechanisms (Braun et al., 2017), and improving treatment that based on biological feedback (Lee et al., 2021). However, beyond its “technical” contributions, our research highlights AI’s potential to increase interpersonal (i.e., one can describe an interpersonal situation and ask ChatGPT to suggest what emotions the other person probably felt) and intrapersonal (i.e., one can describe a situation and ask ChatGPT to suggest what emotions they probably felt) understanding, which is considered a core skill in clinical psychotherapy.

As EA is considered a fundamental ability in psychological practice that moderates the effectiveness of psychodynamic, cognitive behavioral therapies (Virtue et al., 2019), and humanistic psychotherapy (Cain et al., 2016) our findings have valuable theoretical and clinical implications. Clinical populations with EA impairments such as borderline personality disorder, somatoform disorders, eating disorders, PTSD, depression, schizophrenia, and addiction (Lane and Smith, 2021) may use ChatGPT as part of protentional cognitive training to improve their ability to identify and describe their emotions. The combination of ChatGPT’s theoretical knowledge with its ability to identify emotions that can be measured using objective measurements such as the LEAS implies that it could also contribute to psychological diagnosis and assessment. Finally, ChatGPT can be used for evaluating the qualifications of mental health professionals and to enrich their emotional language and professional skills. Considering ChatGPT’s relevance in the context of a direct conversation with the patient, although our research findings show that the ChatGPT can “understand” the emotional state of the other, it is not clear whether a human patient would feel “understood” by its answers, and whether knowing that they are conversing with an AI chatbot and not a human therapist would affect the patient’s sense of comprehensibility. Accordingly, previous studies that discussed therapy through virtual means have suggested expanding the definition of empathy beyond a one-on-one encounter (Agosta, 2018).

ChatGPT showed a significant improvement in LEAS scores, reaching the ceiling in the second examination, indicating great potential for future improvements. There are various plausible reasons that may account for the observed improvement over time. These reasons include a potential update in the software version, increased allocation of resources towards each response, or an enhancement in the chat’s effectiveness resulting from user feedback. Our understanding suggests that ChatGPT improvement was not a consequence of learning from our feedback in the first evaluation, as no feedback was provided. Additionally, it is apparent that the improvement was also evident in the five control scenarios that were not transferred during the first evaluation. New tests/codes should be developed to measure the performance of future ChatGPT versions.

Alan Turing, a key figure in the development of modern computing over 70 years ago, introduced the Turing test to evaluate a machine’s capacity to display intelligent behavior (Moor, 1976). This test involves a human examiner holding a natural language conversation with two concealed entities, a human and a machine. If the examiner cannot confidently differentiate between the two entities, then the machine has passed the test. In relation to this study findings, we hypothesize that if an examiner can identify AI as a machine, it would probably be because humans are unlikely to generate responses of such exceptional quality. This issue again emphasizes the question: what will a human feel upon receiving such an answer from an AI chatbot? Beyond the knowledge that a machine gave the responses, humans may feel the responses do not reflect a human level of EA nor the diversity of the human response.

The current study presents significant potential in its findings; however, several limitations should be acknowledged. First, the interaction with ChatGPT was conducted solely in English, while the norms data used for comparison was collected from a French-speaking general population. This linguistic discrepancy raises concerns about the accuracy and validity of the comparison, as language differences may influence the scores obtained. Nonetheless, it should be noted that the LEAS scores of normal English-speaking samples are similar to the norms of the French-speaking general population (Maroti et al., 2018). The current study chose to use the largest available sample of a general population (*n* = 750), which happened to be in French.

Secondly, ChatGPT has been reported to sometimes provide illusory responses (Dahlkemper et al., 2023). It is easier to identify incorrect answers in knowledge-based interactions, whereas emotionally aware responses are inherently subjective, rendering it challenging to determine their correctness. To address this limitation, the current study enlisted two licensed psychologists to evaluate the ChatGPT’s responses. It is vital to consider this limitation when interpreting the ChatGPT’s EA abilities.

Finally, our study reported on a specific AI model during a specific period; we did not test other large language models such as BLOOM or T5. Therefore, to promote our understanding about the general EA ability of AI, future studies should investigate EA performance in other large language models.

In conclusion, the use of AI in mental health care presents both opportunities and challenges. Concerns around dehumanization, privacy, and accessibility should be addressed through further research to realize the benefits of AI while mitigating its potential risks. Another important consideration is the possible limitation of age and cultural adjustment for AI use. Therefore, future research should aim to develop culturally and age-sensitive AI technologies. In addition, further research will need to clarify how the EA-like responses of ChatGPT can be used and designed for applied psychology. Ultimately, the successful integration of AI in mental health care will require a thoughtful and ethical approach that balances the potential benefits and concerns about data ethics, lack of guidance on development and integration of AI applications, potential misuse leading to health inequalities, respecting patient autonomy, and algorithm transparency (Fiske et al., 2019).

## Data availability statement

The raw data supporting the conclusions of this article will be made available by the authors, without undue reservation.

## Author contributions

ZE: conception and design of the study, acquisition and analysis of data, and drafting of a significant portion of the manuscript and tables. DH-S and KA: conception and design of the study, acquisition and analysis of data, and drafting of a significant portion of the manuscript. ML: conception and design of the study and drafting of a significant portion of the manuscript. All authors contributed to the article and approved the submitted version.

## Conflict of interest

The authors declare that the research was conducted in the absence of any commercial or financial relationships that could be construed as a potential conflict of interest.

## Publisher’s note

All claims expressed in this article are solely those of the authors and do not necessarily represent those of their affiliated organizations, or those of the publisher, the editors and the reviewers. Any product that may be evaluated in this article, or claim that may be made by its manufacturer, is not guaranteed or endorsed by the publisher.

## Footnotes

^1^OpenAI SF. ChatGTP [Internet]. Available from: https://chat.openai.com/chat

## References

[ref1] Agosta L. (2018). “Empathy in cyberspace” in Theory and practice of online therapy. eds. Weinberg H. Rolnick A. (New York, NY: Routledge), 34–46.

[ref2] Bagby R. M. Parker J. D. Taylor G. J. (1994). The twenty-item Toronto alexithymia scale—I. Item selection and cross-validation of the factor structure. J. Psychosom. Res. 38, 23–32. doi: 10.1016/0022-3999(94)90005-1, PMID: 8126686

[ref3] Bar-On R. (2000). “Emotional and social intelligence: insights from the emotional quotient inventory” in The handbook of emotional intelligence: theory, development, assessment, and application at home, school, and in the workplace. eds. Bar-On R. Parker J. D. A. (Hoboken, NJ: Jossey-Bass/Wiley), 363–388.

[ref4] Baron-Cohen S. Wheelwright S. Hill J. Raste Y. Plumb I. (2001). The “Reading the mind in the eyes” test revised version: a study with normal adults, and adults with Asperger syndrome or high-functioning autism. J. Child Psychol. Psychiatry 42, 241–251. doi: 10.1111/1469-7610.00715 11280420

[ref5] Baslet G. Termini L. Herbener E. (2009). Deficits in emotional awareness in schizophrenia and their relationship with other measures of functioning. J. Nerv. Ment. Dis. 197, 655–660. doi: 10.1097/NMD.0b013e3181b3b20f, PMID: 19752644 PMC2779570

[ref6] Benjamini Y. Hochberg Y. (1995). Controlling the false discovery rate: a practical and powerful approach to multiple testing. J. R. Stat. Soc. 57, 289–300. doi: 10.1111/j.2517-6161.1995.tb02031.x

[ref7] Birch S. A. J. Bloom P. (2007). The curse of knowledge in reasoning about false beliefs. Psychol. Sci. 18, 382–386. doi: 10.1111/j.1467-9280.2007.01909.x 17576275

[ref8] Braun U. Schaefer A. Betzel R. F. Tost H. Meyer-Lindenberg A. Bassett D. S. (2017). From maps to multi-dimensional network mechanisms of mental disorders. Neuron 97, 14–31. doi: 10.1016/j.neuron.2017.11.007, PMID: 29301099 PMC5757246

[ref9] Burger A. J. Lumley M. A. Carty J. N. Latsch D. V. Thakur E. R. Hyde-Nolan M. E.. (2016). The effects of a novel psychological attribution and emotional awareness and expression therapy for chronic musculoskeletal pain: a preliminary, uncontrolled trial. J. Psychosom. Res. 81, 1–8. doi: 10.1016/j.jpsychores.2015.12.003, PMID: 26800632 PMC4724386

[ref10] Bydlowski S. Corcos M. Jeammet P. Paterniti S. Berthoz S. Laurier C.. (2005). Emotion-processing deficits in eating disorders. Int. J. Eat. Disord. 37, 321–329. doi: 10.1002/eat.20132, PMID: 15856501

[ref11] Bzdok D. Meyer-Lindenberg A. (2018). Machine learning for precision psychiatry: opportunities and challenges. Biol. Psychiatry Cogn. Neurosci. Neuroimaging. 3, 223–230. doi: 10.1016/j.bpsc.2017.11.007, PMID: 29486863

[ref12] Cain D. J. Keenan K. Rubin S. (2016). Humanistic psychotherapies: Handbook of research and practice 2. Washington, DC: American Psychological AssociationPlaceholder Text

[ref13] Chhatwal J. Lane R. D. (2016). A cognitive-developmental model of emotional awareness and its application to the practice of psychotherapy. Psychodyn. Psychiatry. 44, 305–325. doi: 10.1521/pdps.2016.44.2.305, PMID: 27200467

[ref14] Cicchetti D. V. (1994). Guidelines, criteria, and rules of thumb for evaluating normed and standardized assessment instruments in psychology. Psychol. Assess. 6, 284–290. doi: 10.1037/1040-3590.6.4.284

[ref15] Corp IBM. (2021). IBM SPSS statistics for windows (version 28.0) [computer software] (Version 28.0). Armonk, NY: IBM Corp.

[ref16] Dahlkemper M. N. Lahme S. Z. Klein P. (2023). How do physics students evaluate ChatGPT responses on comprehension questions? A study on the perceived scientific accuracy and linguistic quality. *arXiv*. [Epub ahead of preprint]. 1–20. Available at: 10.48550/arXiv.2304.05906

[ref17] Danieli M. Ciulli T. Mousavi S. M. Silvestri G. Barbato S. Di Nitale L.. (2022). Assessing the impact of conversational artificial intelligence in the treatment of stress and anxiety in aging adults: randomized controlled trial. JMIR Ment. Health. 9:e38067. doi: 10.2196/38067, PMID: 36149730 PMC9547337

[ref18] Davis M. H. (1980). Interpersonal reactivity index (IRI). [Database record]. APA PsycTests. doi: 10.1037/t01093-000

[ref19] Donges U. S. Kersting A. Dannlowski U. Lalee-Mentzel J. Arolt V. Suslow T. (2005). Reduced awareness of others’ emotions in unipolar depressed patients. J. Nerv. Ment. Dis. 193, 331–337. doi: 10.1097/01.nmd.0000161683.02482.19, PMID: 15870617

[ref20] Doraiswamy P. M. Blease C. Bodner K. (2020). Artificial intelligence and the future of psychiatry: insights from a global physician survey. Artif. Intell. Med. 102:101753. doi: 10.1016/j.artmed.2019.101753, PMID: 31980092

[ref21] Fiske A. Henningsen P. Buyx A. (2019). Your robot therapist will see you now: ethical implications of embodied artificial intelligence in psychiatry, psychology, and psychotherapy. J. Med. Internet Res. 21, e13216–e13212. doi: 10.2196/13216, PMID: 31094356 PMC6532335

[ref22] Fitzpatrick K. K. Darcy A. Vierhile M. Darcy A. (2017). Delivering cognitive behavior therapy to young adults with symptoms of depression and anxiety using a fully automated conversational agent (Woebot): a randomized controlled trial. JMIR Ment. Health. 4, e19–e11. doi: 10.2196/mental.7785, PMID: 28588005 PMC5478797

[ref23] Frewen P. Lane R. D. Neufeld R. W. Densmore M. Stevens T. Lanius R. (2008). Neural correlates of levels of emotional awareness during trauma script-imagery in posttraumatic stress disorder. Psychosom. Med. 70, 27–31. doi: 10.1097/PSY.0b013e31815f66d4, PMID: 18158370

[ref24] Happé F. G. (1994). An advanced test of theory of mind: understanding of story characters’ thoughts and feelings by able autistic, mentally handicapped, and normal children and adults. J. Autism Dev. Disord. 24, 129–154. doi: 10.1007/BF02172093, PMID: 8040158

[ref25] Hu K. (2023). ChatGPT sets record for fastest-growing user base—analyst note. Reuters. Available at: https://www.reuters.com/technology/chatgpt-sets-record-fastest-growing-user-base-analyst-note-2023-02-01/

[ref26] Ji Z. Liu Z. Lee N. Yu T. Wilie B. Zeng M.. (2022). RHO (ρ): reducing hallucination in open-domain dialogues with knowledge grounding. *arXiv*. [Epub ahead of preprint]. Available at: 10.48550/arXiv.2212.01588

[ref27] Kung T. H. Cheatham M. Medenilla A. Sillos C. De Leon L. Elepaño C. Madriaga. (2023). Performance of ChatGPT on USMLE: potential for AI-assisted medical education using large language models. PLOS Digit. Health. 2:e0000198, doi: 10.1371/journal.pdig.0000198, PMID: 36812645 PMC9931230

[ref28] Lane R. D. Quinlan D. Schwartz G. Walker M. P. Zeitlin S. B. (1990). The levels of emotional awareness scale: a cognitive–developmental measure of emotion. J. Pers. Assess. 55, 124–134. doi: 10.1080/00223891.1990.9674052 2231235

[ref29] Lane R. D. Smith R. (2021). Levels of emotional awareness: theory and measurement of a socio-emotional skill. J. Intelligence 9:42. doi: 10.3390/jintelligence9030042, PMID: 34449662 PMC8395748

[ref30] Lee E. E. Torous J. De Choudhury M. Depp C. A. Graham S. A. Kim. (2021). Artificial intelligence for mental health care: clinical applications, barriers, facilitators, and artificial wisdom. Biol. Psychiatry Cogn. Neurosci. Neuroimaging. 6, 856–864. doi: 10.1016/j.bpsc.2021.02.001, PMID: 33571718 PMC8349367

[ref31] Levine D. Marziali E. Hood J. (1997). Emotion processing in borderline personality disorders. J. Nerv. Ment. Dis. 185, 240–246. doi: 10.1097/00005053-199704000-00004 9114809

[ref32] Maroti D. Lilliengren P. Bileviciute-Ljungar I. (2018). The relationship between alexithymia and emotional awareness: a meta-analytic review of the correlation between TAS-20 and LEAS. Front. Psychol. 9:453. doi: 10.3389/fpsyg.2018.00453, PMID: 29713295 PMC5911526

[ref33] Montag C. Haase L. Seidel D. Bayerl M. Gallinat J. Herrmann U.. (2014). A pilot RCT of psychodynamic group art therapy for patients in acute psychotic episodes: feasibility, impact on symptoms and mentalising capacity. PLoS One 9:e112348. doi: 10.1371/journal.pone.0112348 25393414 PMC4231093

[ref34] Moor J. H. (1976). An analysis of the Turing test. Philos. Stud. 30, 249–257. doi: 10.1007/BF00372497

[ref35] Nandrino J. L. Baracca M. Antoine P. Paget V. Bydlowski S. Carton S. (2013). Level of emotional awareness in the general French population: effects of gender, age, and education level. Int. J. Psychol. 48, 1072–1079. doi: 10.1080/00207594.2012.753149, PMID: 23305070

[ref36] Neumann D. Malec J. F. Hammond F. M. (2017). Reductions in alexithymia and emotion dysregulation after training emotional self-awareness following traumatic brain injury: a phase I trial. J. Head Trauma Rehabil. 32, 286–295. doi: 10.1097/HTR.0000000000000277, PMID: 28060205 PMC5498277

[ref37] Pham K. T. Nabizadeh A. Selek S. (2022). Artificial intelligence and chatbots in psychiatry. Psychiatry Q. 93, 249–253. doi: 10.1007/s11126-022-09973-8, PMID: 35212940 PMC8873348

[ref38] Radice-Neumann D. Zupan B. Tomita M. Willer B. (2009). Training emotional processing in persons with brain injury. J. Head Trauma Rehabil. 24, 313–323. doi: 10.1097/HTR.0b013e3181b09160 19858965

[ref39] Rudolph J. Tan S. Tan S. (2023). ChatGPT: bullshit spewer or the end of traditional assessments in higher education? J. Appl. Learn. Teach. 6, 1–22. doi: 10.37074/jalt.2023.6.1.9

[ref40] Subic-Wrana C. Bruder S. Thomas W. Lane R. D. Köhle K. (2005). Emotional awareness deficits in inpatients of a psychosomatic ward: a comparison of two different measures of alexithymia. Psychosom. Med. 67, 483–489. doi: 10.1097/01.psy.0000160461.19239.13, PMID: 15911914

[ref41] Topol E. (2019). Deep medicine: how artificial intelligence can make healthcare human again. New York City, NY: Basic Books.

[ref42] Vajawat B. Varshney P. Banerjee D. (2021). Digital gaming interventions in psychiatry: evidence, applications and challenges. Psychiatry Res. 295:113585. doi: 10.1016/j.psychres.2020.113585, PMID: 33303223

[ref43] Virtue S. M. Manne S. L. Criswell K. Kissane D. Heckman C. J. Rotter D. (2019). Levels of emotional awareness during psychotherapy among gynecologic cancer patients. Palliat. Support. Care 17, 87–94. doi: 10.1017/S1478951518000263, PMID: 29880065 PMC8370270

[ref44] Weissman D. G. Nook E. C. Dews A. A. Miller A. B. Lambert H. K. Sasse S. F.. (2020). Low emotional awareness as a transdiagnostic mechanism underlying psychopathology in adolescence. Clin. Psychol. Sci. 8, 971–988. doi: 10.1177/2167702620923649, PMID: 33758688 PMC7983841

